# Identification and Characterization of the Lactating Mouse Mammary Gland Citrullinome

**DOI:** 10.3390/ijms21072634

**Published:** 2020-04-10

**Authors:** Guangyuan Li, Coleman H. Young, Bryce Snow, Amanda O. Christensen, M. Kristen Demoruelle, Venkatesh V. Nemmara, Paul R. Thompson, Heather M. Rothfuss, Brian D. Cherrington

**Affiliations:** 1 Department of Zoology and Physiology, University of Wyoming, Laramie, WY 82071, USA; guangyuanli1010@gmail.com (G.L.); cyoung19@uwyo.edu (C.H.Y.); bsnow1@uwyo.edu (B.S.); achris36@uwyo.edu (A.O.C.); hrothfu1@uwyo.edu (H.M.R.); 2 Division of Rheumatology, University of Colorado School of Medicine, Aurora, CO 80045, USA; Kristen.demoruelle@cuanschutz.edu; 3 Department of Chemistry and Biochemistry, Rowan University, Glassboro, NJ 08028, USA; Nemmara@rowan.edu; 4 Department of Biochemistry and Molecular Pharmacology, University of Massachusetts Medical School, Worcester, MA 01605, USA; Paul.Thompson@umassmed.edu

**Keywords:** peptidylarginine deiminase, citrullination, prolactin, mammary gland, lactation

## Abstract

Citrullination is a post-translational modification (PTM) in which positively charged peptidyl-arginine is converted into neutral peptidyl-citrulline by peptidylarginine deiminase (PAD or PADI) enzymes. The full protein citrullinome in many tissues is unknown. Herein, we used mass spectrometry and identified 107 citrullinated proteins in the lactation day 9 (L9) mouse mammary gland including histone H2A, α-tubulin, and β-casein. Given the importance of prolactin to lactation, we next tested if it stimulates PAD-catalyzed citrullination using mouse mammary epithelial CID-9 cells. Stimulation of CID-9 cells with 5 µg/mL prolactin for 10 min induced a 2-fold increase in histone H2A citrullination and a 4.5-fold increase in α-tubulin citrullination. We next investigated if prolactin-induced citrullination regulates the expression of lactation genes β-casein (*Csn2*) and butyrophilin (*Btn1a1*). Prolactin treatment for 12 h increased β-casein and butyrophilin mRNA expression; however, this increase was significantly inhibited by the pan-PAD inhibitor, BB-Cl-amidine (BB-ClA). We also examined the effect of tubulin citrullination on the overall polymerization rate of microtubules. Our results show that citrullinated tubulin had a higher maximum overall polymerization rate. Our work suggests that protein citrullination is an important PTM that regulates gene expression and microtubule dynamics in mammary epithelial cells.

## 1. Introduction

Multiple peptidylarginine deiminase (PAD or PADI) isoforms are expressed in the mammary glands of mice, dogs, cats, and humans, primarily in luminal epithelial cells [1,2,3]. In dogs and mice, PADs are expressed in lactating mammary gland secretory cells. PAD enzymes remove the positively charged imine group from arginine residues resulting in changes in the structure, function, and molecular interactions of substrate proteins. Histones and cytoskeletal proteins are known substrates for PAD enzymes [4,5,6,7]. In several female reproductive tissues, PADs epigenetically regulate gene expression via citrullination of histone tail arginine residues [2,8,9]. Cytoskeletal proteins are also targets for citrullination, which is thought to alter filament stability and intermolecular interactions [10,11,12]. Citrullinated cytoskeletal filaments are present in the synovial tissue of rheumatoid arthritis (RA) patients and may be involved in disease progression [7,13]. In many tissues, including the mammary gland, the normal physiological function of a majority of citrullinated proteins is unknown.

The peptide hormone prolactin (PRL), secreted from lactotrope cells in the anterior pituitary gland, is required to initiate lactation and milk protein synthesis by mammary secretory cells. PRL binding to its type I cytokine receptor causes receptor dimerization and activation of the Janus kinase 2 (JAK2)/signal transducer and activator of transcription 5 (STAT5) signaling pathway. Phosphorylated STAT5a and STAT5b dimerize, translocate to the nucleus, and target interferon-γ-activated sequence (GAS) motifs on lactation related gene promoters to induce gene transcription by mammary secretory cells [14,15]. Prolactin stimulates robust transcription of β-casein (*Csn2*), a major milk protein [16,17]. Following translation, milk proteins are processed in the endoplasmic reticulum, packaged into secretory vesicles in the Golgi apparatus, transported to the apical region of the lactating mammary secretory cells via a microtubule networks, and then secreted into the alveoli lumen by exocytosis [17,18]. For milk lipid secretion, the small triacylglycerol-rich lipids form larger cytoplasmic lipid droplets (CLDs) that are transported to the apical membrane of mammary secretory cells. CLDs are encapsulated by membrane/protein complexes that are necessary for milk fat globule secretion [19,20]. Although the molecular mechanisms mediating milk fat globule secretion are not well described, the protein butyrophilin (Btn1a1) is essential to regulate the size of the lipid droplets secreted in milk [21].

Microtubules, composed of α- and β-tubulin heterodimers, form hollow cylindrical polymer structures that increase from late pregnancy through lactation in the guinea pig mammary gland [22]. In mammary epithelial cells, microtubules are abundant in the apical region and orient perpendicular to the plasma membrane [23]. Microtubules are necessary to transport milk protein vesicles to the plasma membrane, and their disruption leads to accumulation of casein-containing vesicles in mammary secretory cells [18,24]. Though the function of microtubules in milk lipid secretion is not well characterized, disrupting microtubules also causes accumulation of lipid droplets within mammary secretory cells [19,25]. Intriguingly, other post-translational modifications (PTMs) of α- and β-tubulin such as phosphorylation and acetylation are well known to alter microtubule organization, dynamics, and protein interactions [26]; yet, it is not known how these same parameters are affected by tubulin citrullination.

Using liquid chromatography with tandem mass spectrometry (LC-MS/MS), we identified 107 citrullinated proteins in the lactation day 9 (L9) mouse mammary gland including histone H2A, α-tubulin, and β-casein. Since prolactin is critical for mammary secretory cell function during lactation, we tested if prolactin stimulates citrullination of histone H2A and α-tubulin using the mouse mammary epithelial CID-9 cell line. Prolactin rapidly increased histone H2A and α-tubulin citrullination within 10 min. In terms of functional consequences, citrullination regulated expression of lactation genes *Csn2* and *Btn1a* and altered the overall polymerization rates of microtubules, suggesting a role for this PTM in both the synthesis and secretion of milk. Lastly, our work was the first to show that β-casein was citrullinated in milk from mice and humans, raising additional interesting questions regarding the biological significance of this understudied PTM.

## 2. Results

### 2.1. 107 Citrullinated Proteins Are Present in the Lactation Day 9 (L9) Mouse Mammary Gland

Our previous studies detected multiple citrullinated proteins in the L9 mouse mammary gland using an anti-modified pan-citrulline Western blot; however, this method could not identify the proteins [2]. To identify the citrullinated proteins, an L9 mouse mammary gland was excised, washed in HEPES buffer, and homogenized. Citrullinated proteins from the L9 mouse mammary gland lysate were labelled with biotin-phenylglyoxal (biotin-PG), which selectively modifies citrullinated proteins. As a control, an equal concentration of L9 mouse mammary gland lysate was incubated without biotin-PG. The biotin-PG-labelled citrullinated proteins and control proteins from the same L9 mammary gland lysate were enriched with streptavidin-conjugated agarose beads and eluted by boiling in loading buffer. The samples were then electrophoresed for 10 min on a polyacrylamide gel and stained with Coomassie blue. The resulting proteome bands were cored, trypsin digested, and the proteins identified via LC-MS/MS, conducted by the proteomic and metabolomics core facility at Colorado State University.

**Figure 1:**
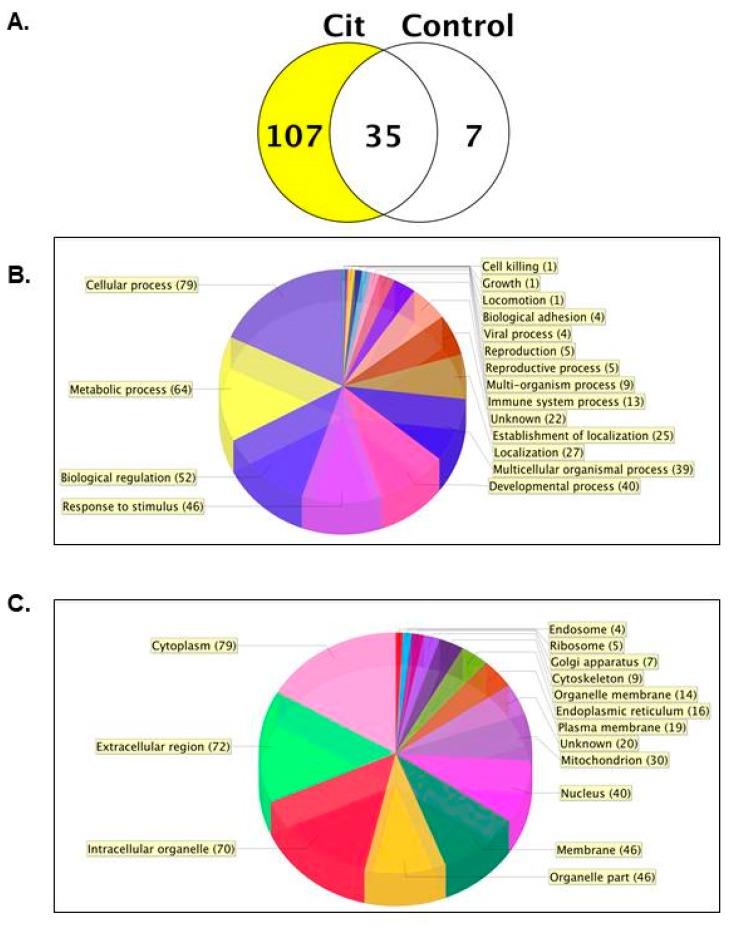
One hundred seven citrullinated proteins were present in the lactation day 9 (L9) mouse mammary gland. (**A**) The Venn diagram shows the number of citrullinated proteins identified with a biotin-PG label (Cit) and without biotin-PG as the negative control (Control) in the L9 mouse mammary gland. The pie charts depict gene ontology (GO) analysis of citrullinated protein distribution at the biological process (**B**) and cellular compartment levels (**C**).

Scaffold identified 107 proteins in the L9 mouse mammary gland that were selectively isolated with biotin-PG. These numbers are based on hits to the reverse database with a set peptide threshold (0.1%) and a false discovery rate (FDR) of 0.08% (Figure 1A and Table S1). The distribution of the citrullinated proteins at the biological process and subcellular level were examined using gene ontology (GO) analysis. Sixty-four citrullinated proteins were associated with metabolic processes such as regulation of amino acids, glucose, carbohydrates, and lipids, which suggests the potential physiological importance of citrullinated proteins in milk production (Figure 1B). In terms of subcellular localization, 79 cytoplasmic proteins were citrullinated, 9 of which were cytoskeletal, while 40 citrullinated proteins were nuclear (Figure 1C).

**Figure 2:**
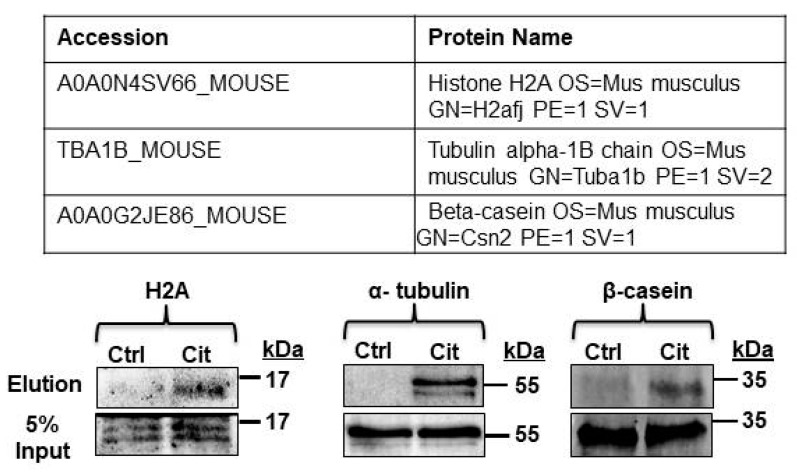
Histone H2A, α-tubulin, and β-casein were citrullinated in the L9 mouse mammary gland. L9 mouse mammary glands were harvested in HEPES buffer, then equal concentrations of lysate were labeled with biotin-PG (Cit) or without the probe as the negative control (Ctrl). Citrullinated proteins were enriched with streptavidin-conjugated agarose beads. Enriched citrullinated proteins were then subjected to Western blot analysis with anti-histone H2A, α-tubulin, or β-casein antibodies. A 5% input sample was collected before enrichment and served as the loading control.

Based on these results, we chose to validate the citrullination of proteins from distinct subcellular compartments in L9 mammary glands. These proteins include histone H2A, α-tubulin, and the major milk protein β-casein. Total citrullinated proteins were enriched with biotin-PG or with vehicle as a negative control following the same experimental paradigm described above. Citrullinated protein samples were examined by Western blots and probed with anti-histone H2A, α-tubulin, and β-casein antibodies. Then, 5% of the input sample was removed prior to enrichment and served as the loading control. Western blot analysis validated that citrullinated histone H2A, α-tubulin, and β-casein were present in L9 mouse mammary gland (Figure 2). Still at issue is the mechanism initiating citrullination and ultimately its effect on protein function.

### 2.2. Prolactin Induces Histone H2A Citrullination in Mouse Mammary Epithelial CID-9 Cells

**Figure 3:**
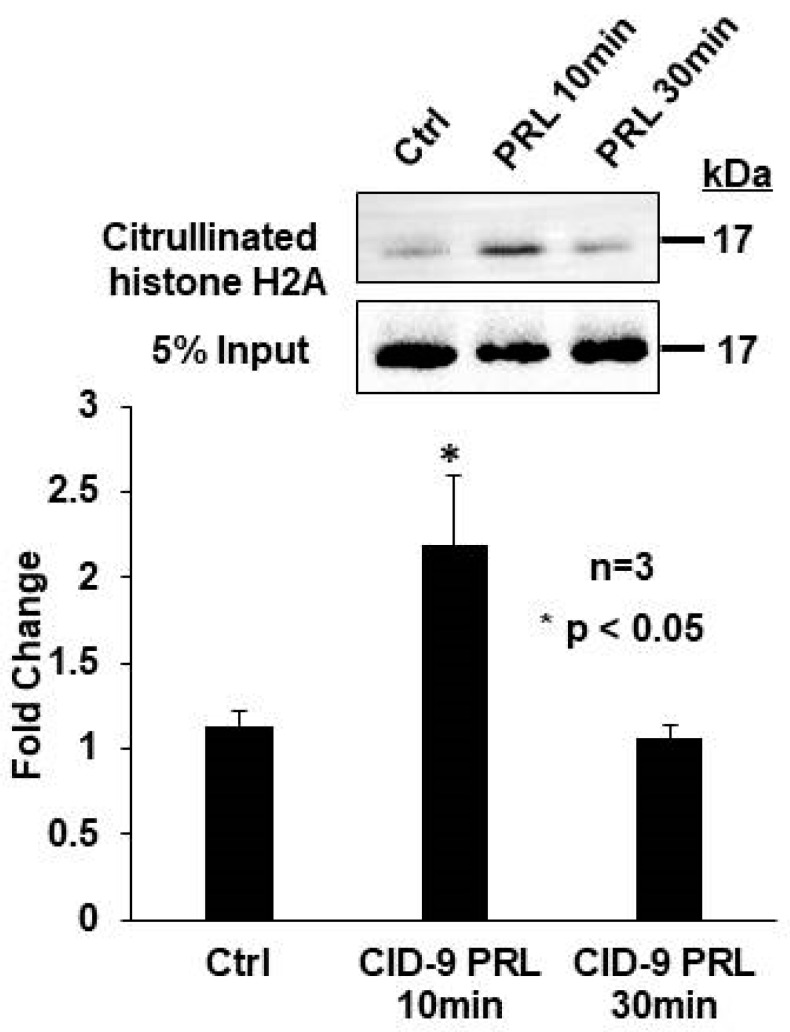
Prolactin induced histone H2A citrullination in mouse mammary epithelial CID-9 cells. CID-9 cells were treated with vehicle or 5 µg/mL prolactin for 10 and 30 min. Equal amounts of lysate from each time point were labeled with biotin-PG and enriched with streptavidin-conjugated agarose beads. A 5% input sample was removed before enrichment and served as the loading control. Citrullinated histones and 5% input samples were examined by Western blot analysis, and membranes were probed with the anti-histone H2A antibody. The top panel shows a representative Western blot, while the bottom panel represents the quantification of multiple Western blots using BioRad Image Lab 4.0. Data are presented as means +/− SEM and separated using ANOVA SNK (*n* = 3, * *p* < 0.05).

We next hypothesized that prolactin stimulates histone H2A citrullination to initiate milk gene expression. To test our hypothesis, CID-9 mouse mammary epithelial cells, which express PADs and are prolactin responsive, were treated with vehicle or 5 µg/mL prolactin for 10 or 30 min. Following treatment, cells were lysed and equal concentrations of lysate from each time point were labeled with biotin-PG, while 5% input was removed prior to enrichment and served as a loading control. Resulting samples and 5% input controls were examined by Western blot, and membranes were probed with an anti-histone H2A antibody. A representative Western blot and quantification of multiple blots revealed that 10 min stimulation of CID-9 cells with 5 µg/mL prolactin significantly increased citrullination of histone H2A by greater than 2-fold (Figure 3). These results suggest that prolactin stimulates histone H2A citrullination; however, the genes regulated by this mechanism are unknown.

### 2.3. Prolactin Stimulates Citrullination to Increase β-Casein and Butyrophilin mRNA Expression in CID-9 Cells

**Figure 4:**
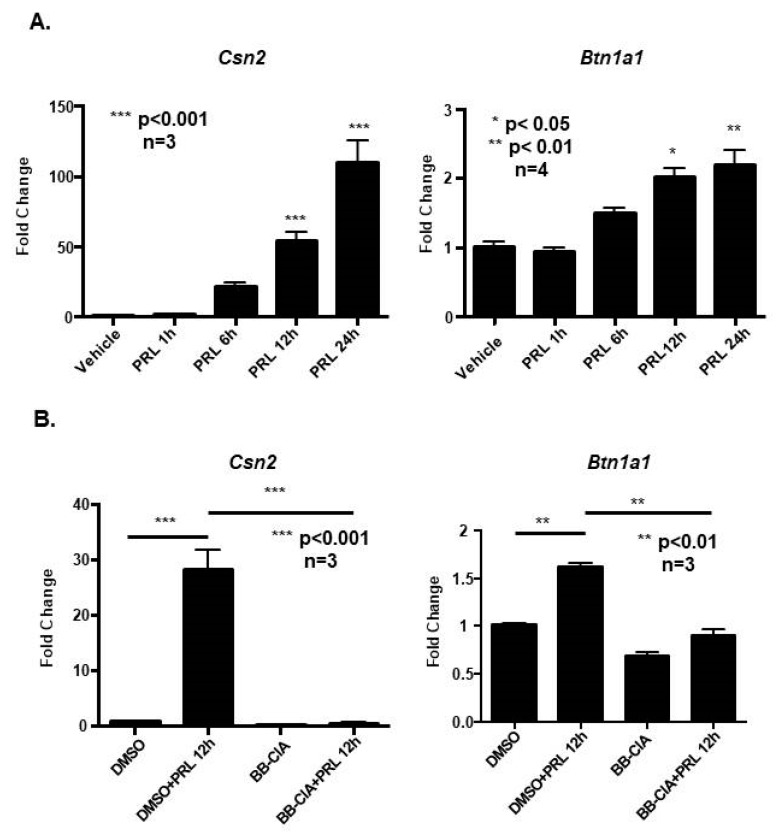
Inhibiting citrullination blocked prolactin induced expression of milk proteins β-casein and butyrophilin in mouse mammary epithelial CID-9 cells. (**A**) CID-9 cells were treated with vehicle or 5 µg/mL prolactin for 1, 6, 12, or 24 h. Total RNA was extracted, reverse transcribed, and subjected to qPCR performed with primers specific to Csn2 and Btn1a1 or Gapdh as the endogenous controls (*n* < 4, * *p* < 0.05, ** *p* < 0.01, *** *p* < 0.001). (**B**) CID-9 cells were pre-treated with vehicle or 2 µM of BB-ClA for 1 h, followed by 5 µg/mL prolactin treatment for 12 h. Total RNA was extracted, reverse transcribed, and subjected to qPCR performed with primers specific to Csn2 and Btn1a1 or Gapdh as the endogenous control (*n* = 3, ** *p* < 0.01, *** *p* < 0.001). Data are presented as means +/− SEM and separated using ANOVA SNK.

Since it is well documented that histone citrullination alters gene expression, we next tested if lactation-related genes are regulated by prolactin-induced citrullination in CID-9 cells. To determine a time course for lactation-related mRNA expression, CID-9 cells were first treated with vehicle or 5 µg/mL prolactin for 1, 6, 12, and 24 h. Following cell lysis and RNA purification, samples were subjected to qPCR analysis with specific primers to the milk mRNAs β-casein and butyrophilin. Results indicate that prolactin stimulation of CID-9 cells for 12 h significantly increases β-casein and butyrophilin mRNA expression by approximately 50-fold and 2-fold, respectively, compared to vehicle treated cells (Figure 4A). We next tested if inhibiting PAD activity blunts prolactin induced expression of the β-casein and butyrophilin mRNA. CID-9 cells were pre-treated with DMSO or 2 μM BB-ClA for 1 h, followed by treatment with vehicle or 5 µg/mL prolactin for 12 h. While 12 h prolactin treatment significantly increased β-casein and butyrophilin mRNA, pre-treatment of CID-9 cells with BB-ClA significantly blocked prolactin induced expression of these mRNAs (Figure 4B). Our data suggest that β-casein and butyrophilin expression is regulated by prolactin-induced citrullination in CID-9 cells.

### 2.4. Prolactin Stimulates Citrullination of α-Tubulin in CID-9 Cells

**Figure 5:**
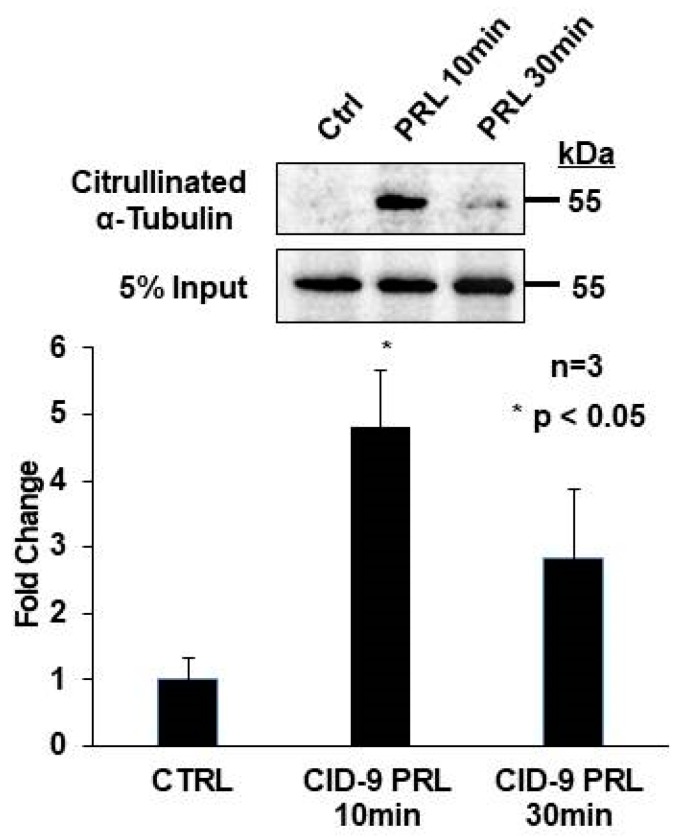
Prolactin stimulated α-tubulin citrullination in mouse mammary epithelial CID-9 cells. CID-9 cells were treated with vehicle or 5 µg/mL prolactin for 10 and 30 min. Equal amounts of lysate from each time point were labeled with biotin-PG and enriched with streptavidin-conjugated agarose beads. A 5% input sample was removed before enrichment and served as the loading control. Citrullinated histones and 5% input samples were examined by Western blot analysis, and membranes were probed with the anti-α-tubulin antibody. The top panel shows a representative Western blot, while the bottom panel represents the quantification of multiple Western blots using BioRad Image Lab 4.0. Data are presented as means +/− SEM and separated using ANOVA SNK (*n* = 3, * *p* < 0.05).

The α-tubulin was also citrullinated in L9 mouse mammary glands, and mass spectrometry analysis revealed a high number of peptide hits. Interestingly, citrullinated tubulin is present in synovial tissue from RA patients [27]; however, the physiological function of citrullinated tubulin in mammary epithelial cells is unknown. Therefore, we chose to investigate if prolactin stimulates α-tubulin citrullination in CID-9 cells. To test this, CID-9 cells were treated with vehicle or 5 µg/mL prolactin for 10 or 30 min then harvested in HEPES buffer. Lysates were labeled with biotin-PG, and the citrullinated proteins were enriched. Following washing and elution, citrullinated proteins and 5% input were analyzed by Western blot, and membranes were probed with an anti-α-tubulin antibody. A representative Western blot and quantification of multiple blots reveals that 10 min of 5 µg/mL prolactin treatment induced a significant 4.5-fold increase in α-tubulin citrullination (Figure 5). Our result show for the first time that prolactin stimulated citrullination of α-tubulin in mammary epithelial cells.

### 2.5. Citrullination Alters Tubulin Polymerization Rates

**Figure 6:**
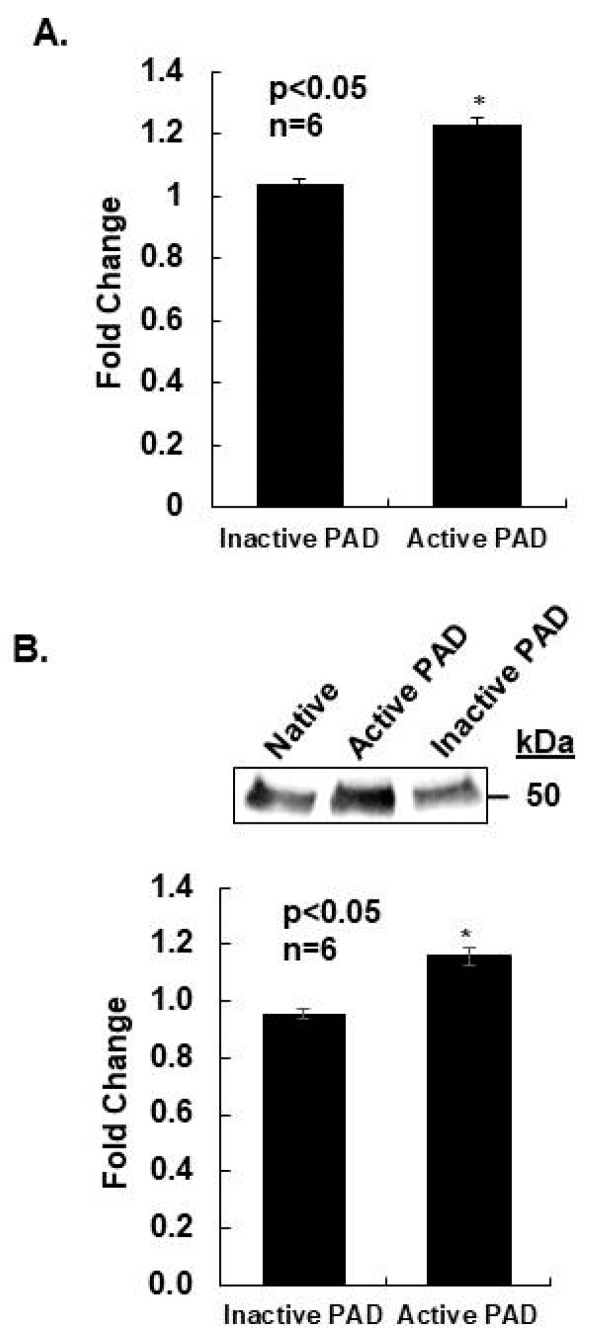
Citrullination altered tubulin polymerization rates. (**A**) Purified porcine brain tubulin was incubated with vehicle (native), purified PAD enzyme with calcium (active PAD), or purified PAD enzyme without calcium (inactive PAD). Native, active PAD, and inactive PAD tubulin samples were polymerized into microtubules and rates measured every 20 s through the entire polymerization range until steady-state equilibrium was reached. The tubulin polymerization Vmax was calculated as the maximum 5 min average slope of polymerization for all three samples. The active PAD and inactive PAD Vmax values are represented as fold change compared to native tubulin Vmax. All values are expressed as the mean ± SEM. Means were separated using Student’s *t*-test and * indicates significantly different means (*n* = 6, * *p* < 0.05). (**B**) After completion of the polymerization assay, 4 µgs of native, active PAD, and inactive PAD tubulin samples were labelled with biotin-PG and examined by Western blot. The top panel shows a representative Western blot, while the bottom panel represents the quantification of multiple Western blots using ThermoFisher iBright Analysis software. Densitometry results for citrullinated tubulin for active PAD and inactive PAD samples were normalized to native tubulin to allow for comparison across different experiments. Data are presented as means +/− SEM and separated using the student Student’s *t*-test (*n* = 6, * *p* < 0.05).

PTMs alter the structure and function of tubulin, but the effect of citrullination has not been described. To begin to address this interesting question, we examined the effect of PAD catalyzed in vitro citrullination of purified tubulin on polymerization rates. First, 100 μgs of purified porcine brain tubulin was incubated with vehicle (native), purified PAD enzyme with calcium (active PAD), or purified PAD enzyme without calcium (inactive PAD). Polymerization rates were then measured using a tubulin polymerization assay and active and inactive PAD tubulin samples were normalized to rates for native tubulin [28,29,30]. Our results show that in vitro citrullination of tubulin increased Vmax 1.2-fold compared to control (Figure 6A). Polymerization rates of native and inactive PAD samples were not significantly different, suggesting that tubulin citrullination, not PAD binding, is responsible for the increased rate of polymerization. Following polymerization assays, 4 μgs of tubulin from each sample (native, active PAD, inactive PAD) were labelled with biotin-PG, examined by Western blot, and quantified using densitometry. Citrullination levels for active and inactive PAD tubulin samples were normalized to native tubulin. Interestingly, native tubulin was citrullinated, but incubation with active PAD enzyme increased levels by approximately 16% compared to inactive PAD controls (Figure 6B). As expected, citrullinated tubulin levels in the native and inactive PAD samples were not significantly different. Collectively, our results are the first to suggest that PAD-catalyzed citrullination of tubulin increases overall polymerization rates.

### 2.6. β-Casein Is Citrullinated in Mouse and Human Milk

**Figure 7:**
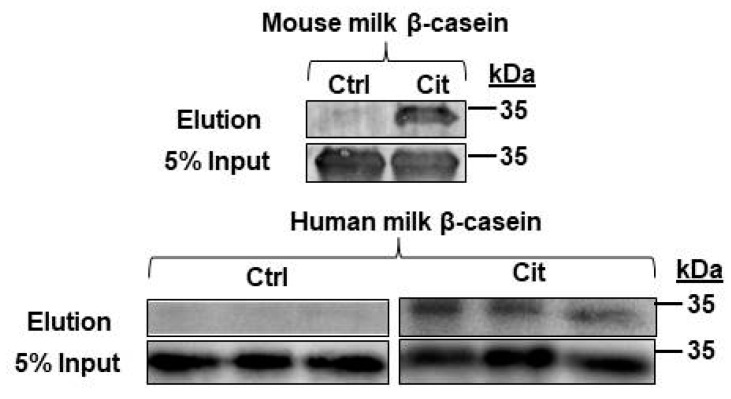
β-casein was citrullinated in mouse and human milk. L9 mouse milk and three human breast milk samples were labelled with biotin-PG (Cit) or without biotin-PG (Ctrl), enriched with streptavidin beads, and then separated by Western blot. PVDF membranes containing enriched citrullinated proteins were then probed with anti-β-casein antibodies. A 5% input sample was collected before enrichment and served as the loading control.

Mass spectrometry and subsequent validation also revealed that the milk protein β-casein was citrullinated in the L9 mouse mammary gland. This finding prompted us to investigate if citrullinated β-casein is present in mouse and human milk. Mouse milk was collected as previously described and three samples were obtained from a human milk bank [31]. Citrullinated proteins in milk were labelled with biotin-PG and enriched with streptavidin conjugated agarose beads. Following washing and elution, biotin-PG-labelled citrullinated proteins and 5% unlabeled input milk were analyzed by Western blot, and membranes were probed with anti-β-casein antibodies. Our results show that citrullination of β-casein was present and conserved between mouse and human milk (Figure 7).

## 3. Discussion 

The physiological function of protein citrullination in the lactating mammary gland has not previously been well defined. This is a significant gap in knowledge given that breast milk is the primary source of nutrition and immunological protection for newborns and since lactation has numerous health benefits for nursing mothers [32,33]. Herein, we address this gap in knowledge by first identifying the citrullinome of the lactating mouse mammary gland and then investigating the function of specific citrullinated proteins. Although identification of different tissue citrullinomes is increasing, our understanding of the consequences of this PTM on protein function remains an important unresolved question.

To identify the citrullinated proteins, we used biotin-PG, which covalently interacts with the urea group of citrulline residues in proteins under acidic conditions [34]. This approach has been used previously to identify more than 50 citrullinated proteins including vimentin, RNA helicases, and nucleophosmin in the HEK293T cell line and more than 150 citrullinated proteins in RA serum, synovial fluid, and synovial tissue samples [7,34]. Our proteomic analysis identified 107 citrullinated proteins including known PAD substrates such as α-enolase and keratins in the L9 mouse mammary gland. We chose to validate the citrullination of histone H2A, α-tubulin, and β-casein because they have distinct subcellular localizations and functions in mammary epithelial cells. A limitation of our approach is that we have identified the citrullinome from the entire lactating mammary gland including milk. Therefore, we cannot conclusively determine the cellular origin of the citrullinated proteins despite the fact that PADs are primarily expressed in the secretory epithelial cells of the lactating mouse mammary gland [1,35].

Citrullinated histone H2A is present in L9 mouse mammary glands, and prolactin rapidly stimulates a significant increase in histone H2A citrullination in CID-9 cells. PAD catalyzed citrullination of histone H2A was first identified in granulocyte-differentiated HL-60 cells, where citrullination occurs at arginine 3 of the N-terminal sequence Ac-SGRGK [36]. In CID-9 cells, Csn2 mRNA expression is stimulated by 12-h prolactin treatment, but it is significantly decreased following pre-treatment with the pan-PAD inhibitor, BB-ClA. Prolactin also stimulates other histone modification to facilitate β-casein gene expression. Kabotyanski et al. showed that prolactin stimulation recruits STAT5 and histone deacetylase 1 (HDAC1) to the *Csn2* promoter [37]. In the lactating mouse mammary gland there is also enrichment of euchromatin markers such as dimethylated lysine 4 histone H3 (H3K4 me2) and histone H3 acetylation (H3Ac) on the *Csn2* promoter [38,39]. Given these findings, it seems likely that prolactin stimulates multiple epigenetic modifications including histone citrullination to upregulate *Csn2* gene expression. Compared to β-casein, much less is known about the expression of butyrophilin despite its important role in milk lipid secretion. Btn1a1 mRNA expression increases more than 2-fold at parturition in mouse mammary gland [17]. The 5′- flanking region of the mouse *Btn1a1* promoter contains several putative STATs binding sites, suggesting a mechanism for prolactin regulation [40]. There is currently no commercial antibody to citrullinated histone H2A. Without this antibody, we are unable to perform chromatin immunoprecipitation to test for a direct association between citrullinated histone H2A and the *Csn2* or *Btn1a1* gene promoters.

Our studies detected citrullinated α-tubulin in the lactating mouse mammary gland and show that 10 min of 5 µg/mL prolactin treatment increases citrullination of α-tubulin compared to vehicle-treated CID-9 cells. Commercially available purified tubulin from porcine brain tissue is highly citrullinated, indicating an important role for this PTM in vivo. To the best of our knowledge, our work is the first to show that in vitro citrullination of tubulin alters overall polymerization kinetics. The relatively small change in Vmax associated with in vitro citrullination of tubulin may be, in part, due to the high endogenous level of citrullination on the native tubulin. The polymerization Vmax of native tubulin is not significantly different in the presence of inactive PAD enzyme, suggesting that the effect is not due to PAD binding to tubulin, but rather citrullination of tubulin. It is likely that citrullination acts in concert with other tubulin PTMs such as acetylation, which can stabilize microtubules and promote binding of molecular motors [26,41]. In lactating mammary epithelial cells, post-Golgi protein-containing vesicles are transported along polymerized microtubules to the apical region of the cell for eventual secretion [24]. Given this, it is possible that citrullination of tubulin not only alters polymerization rates, but also directs molecular motors to traffic milk vesicles along microtubule networks. Regardless, our work suggests that citrullination of tubulin may represent a novel mechanism to facilitate milk vesicle secretion in lactating mammary secretory cells. To better address these intriguing questions, studies are currently underway that will use purified human tubulin from a non-mammalian source which lacks PAD enzymes to circumvent high endogenous levels of citrullination on purified native tubulin.

Equally intriguing, our work for the first time shows that β-casein is citrullinated in mouse and human milk. Currently, the functional significance of this observation is unclear, but caseins contain numerous PTMs. For example, α- and β–caseins are phosphorylated while κ-casein is glycosylated, and these modifications are believed to be important for micelle formation and stability [42]. Micelle structure is an important physical property of milk and provides a delivery mechanism for amino acids and calcium for infant nutrition. Therefore, it will be very interesting to determine if citrullination of β-casein alters micelle structure.

In conclusion, we have identified the citrullinome of the L9 mouse mammary gland and characterized the role of citrullinated histone H2A and α-tubulin in CID-9 mouse mammary epithelial cells. Our work shows that prolactin stimulates *Csn2* and *Btn1a1* expression via citrullination. Simultaneously, prolactin stimulates citrullination of α-tubulin to potentially aid in milk secretion. We propose a model in which PAD-catalyzed citrullination is required for both the synthesis of milk proteins and also for transport of milk vesicles along microtubule networks. Overall, our work advances knowledge of PAD enzyme function in the synthesis and secretion of milk components during lactation.

## 4. Materials and Methods 

### 4.1. Cell Culture and PAD Inhibitor Treatments

The CID-9 cell line was obtained from Dr. Mina Bissell and maintained as previously described [2,43]. For prolactin treatment, CID-9 cells were grown in phenol red free DMEM (Hyclone, Logan, UT, USA) with 2% charcoal stripped FBS (Corning, Mediatech, Manassas, VA, USA), 5 µg/mL insulin (MilliporeSigma, Burlington, MA, USA), and 1 µg/mL hydrocortisone (MilliporeSigma) for 48 h. Cells were then treated with vehicle or 5 µg/mL prolactin (MilliporeSigma) for the designated times. Dr. Paul Thompson synthesized and generously provided the biotin-phenylglyoxal (biotin-PG) probe and the pan-PAD inhibitor, biphenyl-benzimidazole-Cl-amidine (BB-ClA) [34,44]. Cells were pre-treated with vehicle or 2 μM BB-ClA for 1 h prior to prolactin treatments.

### 4.2. Mouse Mammary Tissue and Human Breast Milk

FVB mice were maintained on a 12 h light/dark cycle with ad libitum access to food and water. The abdominal mammary glands were collected from female mice on lactation day 9 as was milk. Euthanasia was performed by CO_2_ asphyxiation and tissue harvested in accordance with the guidelines outlined in the Report of the AVMA on Euthanasia. All work in this study was approved by the University of Wyoming Institutional Animal Care and Use Committee (protocol #20140228BC00067-03, approval 27 February 2017). De-identified human breast milk samples were obtained from the Rocky Mountain Children’s Health Foundation Mother’s Milk Bank (Arvada, CO, USA).

### 4.3. Biotin-Phenylglyoxal Enrichment of Citrullinated Proteins

L9 mouse mammary glands were excised, washed 3× with PBS, and homogenized in 50 mM cold HEPES, pH 7.6. The sample was cleared by centrifugation at 17,000× *g* for 15 min at 4 °C, and the fat layer was removed and the step was repeated until no visible fat layer was observed. Protein concentration was measured by BCA Assay and 300 µg of lysate was incubated with biotin-PG as previously described [34]. Before biotin-PG enrichment, 5% of sample was removed to serve as a loading control. The remaining sample was diluted in 200 μL of PBS containing 0.2% SDS. Then, 100 μL of high-capacity streptavidin or captavidin beads (Thermo Fisher Scientific Inc., Waltham, MA, USA) were added to samples and rotated gently overnight at 4 °C. The bead–protein complexes were washed, and then proteins eluted from the beads by boiling in loading buffer (50 μL; 125 mM Tris-HCL, 10% glycerol, 6 mM EDTA 10% SDS, 8 M urea, and 200 mM DTT) at 100 °C for 15 min. A similar protocol was used to enrich citrullinated proteins from CID-9 cells and milk. Briefly, CID-9 cells were washed with cold PBS, scraped from the plate, pelleted, and then sonicated in 50 mM cold HEPES, pH 7.6. For CID-9 cells and human and mouse milk, protein concentration was measured by BCA Assay and 10 µg of total protein was labeled with biotin-PG as described above. Citrullinated proteins from CID-9 cells and human and mouse milk were examined by Western blot as described below.

### 4.4. LC-MS/MS Proteomics

Citrullinated and control samples were generated as described above using the same L9 mouse mammary gland lysate. The control contained an equal concentration of lysate treated with vehicle rather than biotin-PG. Both citrullinated and control samples were subsequently incubated with streptavidin beads. Following enrichment, lysates were subjected to SDS-PAGE using a 10% gel (acrylamide/bis-acrylamide ratio of 29:1) for 10 min. The gel was then stained with 200 mL Kang’s colloidal Coomassie (0.02% CBB G-250, 5% aluminum sulfate-(14-18)-hydrate, 10% ethanol (96%), and 2% orthophosphoric acid (85%)) overnight on a shaker at room temperature [45]. The next morning, the Coomassie solution was removed and the gel was de-stained with 200 mL destaining solution (10% ethanol (96%) and 2% orthophosphoric acid (85%)) followed by 2 rinses with nanopure water. The proteome bands from the L9 mammary glands and control were cored and subjected to LC-MS/MS proteomic analysis at the Colorado State University Proteomics and Metabolomics Core Facility.

#### 4.4.1. Sample Preparation: In-Gel Trypsin Digestion

Gel fragments were subjected to in-gel trypsin digestion and LC-MS/MS as previously described [46]. Briefly, the gel pieces were washed with 200 μL of LC-MS-grade water (Optima LC-MS, Thermo Fisher Scientific Inc.) for 30 s and destained with 2 × 200 μL of 50% Acetonitrile (ACN; Optima LC-MS Grade)/50 mM Ammonium bicarbonate at 60 °C, with intermittent mixing. The pieces were dehydrated with 100% ACN and allowed to air dry. Proteins were reduced and alkylated in-gel with 25 mM DTT in 50 mM ammonium bicarbonate (60 °C for 20 min) and 55 mM IAA or IAH in 50 mM ammonium bicarbonate at room temperature in the dark for 20 min. Gel pieces were then washed with Optima water and dried. The dried gel pieces were rehydrated in 20 μL 12 ng/μL MS-grade trypsin (Thermo Fisher Scientific Inc.)/0.01% ProteaseMAX surfactant/50 mM ammonium bicarbonate mixture for 10 min at room temperature, overlaid with 30 μL 0.01% ProteaseMAX surfactant/50 mM ammonium bicarbonate, and incubated at 50 °C for 1 h. Extracted peptides were transferred and the digestion halted by addition of 10% trifluoro-acetic acid to a final concentration of 0.5%. Peptide extracts were dehydrated then resuspended in 20 μL of 5% ACN/0.1% formic acid. Once resolubilized, absorbance at 205 nm was measured on a NanoDrop (Thermo Fisher Scientific Inc.) and total peptide concentration was subsequently calculated using an extinction coefficient of 31 [47].

#### 4.4.2. Mass Spectrometry Analysis

A total of ~0.85 μg of peptides were purified and concentrated using an on-line enrichment column (Waters Symmetry Trap C18 100Å, 5 μm, 180 μm ID × 20 mm column). Subsequent chromatographic separation was performed on a reverse phase nanospray column (Waters, Peptide BEH C18; 1.7 μm, 75 μm ID × 150 mm column, 45 °C) using a 90 min gradient: 5–30% buffer B over 85 min followed by 30–45%B over 5 min (B: 0.1% formic acid in ACN) at a flow rate of 350 nanoliters/min. Peptides were eluted directly into the mass spectrometer (Orbitrap Velos, Thermo Fisher Scientific Inc.) equipped with a Nanospray Flex ion source and spectra were collected over a *m*/*z* range of 400–2000, positive mode ionization, using a dynamic exclusion limit of 2 MS/MS spectra of a given *m*/*z* value for 30 s (exclusion duration of 90 s). The instrument was operated in FT mode for MS detection (resolution of 60,000) and ion trap mode for MS/MS detection with normalized collision energy set to 35%. Compound lists of the resulting spectra were generated using Xcalibur 3.0 software (Thermo Fisher Scientific Inc.) with an S/N threshold of 1.5 and 1 scan/group.

#### 4.4.3. Data Analysis

Tandem mass spectra were extracted, charge state deconvoluted, and deisotoped by ProteoWizard MsConvert (version 3.0). Spectra from all samples were searched using Mascot (Matrix Science, London, UK; version 2.6.0) against the Uniprot_Mouse_rev_072017 database with 119,162 entries assuming the digestion enzyme trypsin. Mascot was searched with a fragment ion mass tolerance of 0.80 Da and a parent ion tolerance of 20 PPM. Oxidation of methionine, carboxymethyl of cysteine, and biotin-PG w/Cit of arginine were specified in Mascot as variable modifications. Search results from all samples were imported and combined using the probabilistic protein identification algorithms implemented in the Scaffold software (version Scaffold_4.8.4, Proteome Software Inc., Portland, OR, USA) [48,49]. Peptide thresholds were set (0.1%) such that a peptide FDR of 0.08% was achieved based on hits to the reverse database [50]. Protein identifications were accepted if they could be established at greater than 99.0% probability (≤1% FDR) and contained at least two identified peptides. Protein probabilities were assigned by the Protein Prophet algorithm [51]. Proteins that contained similar peptides and could not be differentiated based on MS/MS analysis alone were grouped to satisfy the principles of parsimony.

#### 4.4.4. Instrument Suitability

Instrument suitability was monitored through analysis of commercially purchased BSA standard digest and automated monitoring using PanormaQC. Metrics (e.g., mass accuracy, peak area, retention time, etc.) were monitored and flagged as outliers if results were outside +/− 3 standard deviations of the guide set (i.e., optimal operation). Values for all metrics were within normal limits throughout the duration of the experiment, indicating instrument stability and data robustness.

### 4.5. Western Blotting

Before biotin-PG enrichment, 5% of L9 mammary gland and CID-9 cell lysates were removed to serve as an input loading control. Samples were subjected to SDS-PAGE using a 12% or 15% gel (acrylamide/bis-acrylamide ratio of 29:1) and subsequently transferred to Immobilon PVDF membranes (MilliporeSigma). Membranes were blocked in 1× casein (Vector Labs, Burlingame, CA, USA) and diluted in Tris buffered saline containing 0.1% Tween-20 (TBS-T) overnight at 4 °C. Primary antibodies were incubated overnight at 4 °C: anti-Histone H2A (Cell Signaling Technology, #12349, Danvers, MA, USA), anti-α-tubulin (Abcam, ab52866, Cambridge, MA, USA), anti-mouse β-casein (Santa Cruz Biotechnology, sc-166530, Dallas, TX, USA), and anti-human β-casein (Abcam, ab205301) were all diluted 1:1000 and incubated overnight at 4 °C. The following morning, membranes were washed in TBS-T, followed by a 2 h incubation at room temperature with 1:10,000 goat anti-rabbit HRP (Jackson ImmunoResearch Labs, West Grove, PA, USA) secondary antibody. All blots were washed for 50 min (5 × 10 min) with TBS-T after secondary antibody incubation and then visualized using SuperSignal West Pico and Femto chemiluminescence substrate (Thermo Fisher Scientific Inc.). Quantitative densitometry analysis was conducted with Bio-Rad Image Lab software (Hercules, CA, USA). Experiments were repeated at least three times. Means were separated using student–Newman–Keuls (SNK) ANOVA and * indicates significantly different means *p* < 0.05.

### 4.6. qPCR

RNA was purified from CID-9 cells as previously described [2]. First, 1 µg of resulting RNA was reverse transcribed using iScript Reverse Transcription Supermix for RT-qPCR (Bio-Rad). Complementary DNA was subject to real time PCR analysis with SYBR Green assays (Bio-Rad) using intron spanning primers specific for Csn2, Btn1a1, or Gapdh as the reference gene control. Csn2 F: 5′ GCAGGCAGAGGATGTGCTC 3′, R: 5′ GAGCATATGGAAAGGCCT 3′; Btn1a1 F: 5′ GCCGCCGTATACCTCAAA 3′, R: 5′ TCCATTCTCTTGAACCGTCA 3′; Gapdh F: 5′ GGGTTCCTATAAATACGGACTGC 3′, R: 5′ CCATTTTGTCTACGGGACGA 3′. Data were analyzed using the delta/delta Ct method in which Ct values of all target genes are adjusted to corresponding Ct value of reference gene Gapdh. All values are expressed as the mean ± SEM. Means were separated using SNK ANOVA and * indicates significantly different means *p* < 0.05.

### 4.7. In Vitro Tubulin Citrullination and Polymerization

Purified porcine brain tubulin was purchased from MP Biomedicals (Irvine, CA, USA), PurSolutions (Nashville, TN, USA), and Cytoskeleton Inc. (Denver, CO, USA). For each experiment, four tubulin samples were prepared in duplicate: negative control, native tubulin, active PAD, and inactive PAD. Samples were resuspended as directed except that 2 mM Ca^2+^ was added to all samples to inhibit tubulin polymerization while simultaneously activating the PAD enzyme for in vitro citrullination [28,29,30]. Then, 1 µL of rabbit skeletal muscle PAD enzyme (Sigma-Aldrich, St. Louis, MO, USA) was added per 100 µg of tubulin to the active PAD sample while the rest received vehicle, and the samples were incubated at 37 °C for 30 min. The negative control sample contained 1 mM Ca^2+^ and served as the negative control for the polymerization assay. EGTA was added to a final concentration of 10 mM in the native tubulin, active PAD, and inactive PAD samples. Free Ca^2+^ after EGTA addition was calculated to be 0.15 μM, sufficiently low to allow for tubulin polymerization and to decrease PAD activity by approximately 6 orders of magnitude [52,53]. After addition of EGTA, 1 µL of rabbit skeletal muscle PAD enzyme per 100 µg tubulin was added to the inactive samples to control for PAD binding.

A tubulin polymerization assay (Cytoskeleton Inc., Denver, CO, USA) was used as previously described [28,29,30]. Briefly, the polymerization reaction was initiated by transfer of negative control, native tubulin, active PAD, and inactive PAD samples in duplicate to a fluorescence plate pre-warmed to 37 °C. Polymerization was monitored at 20 s intervals on a Biotek Synergy 4 96-well fluorescence plate reader with excitation/emission at 350 nm/450 nm. The reactions were monitored through the nucleation, polymerization, and into steady-state equilibrium phases of microtubule polymerization. The Vmax of microtubule formation was determined by calculating the maximum 5 min average slope during polymerization for all samples in each experiment. Vmax values for active PAD and inactive PAD samples were normalized to the native tubulin Vmax to allow for comparison across different experiments. All values are expressed as the mean ± SEM. Means were separated using student’s *t*-test and * indicates significantly different means *p* < 0.05.

#### 4.7.1. Citrullinated Tubulin Western Blot

Post polymerization, 4 µgs of tubulin samples were labelled with biotin-PG and electrophoresed on polyacrylamide gel and transferred to a PVDF membrane as described above. The membrane was then probed with DyLight 488 streptavidin (Vector Labs) to detect citrullinated tubulin. After washing, bands were visualized on an iBright FL1500 Imager (Thermo Fisher Scientific Inc.) made available by the Wyoming Sensory Biology COBRE award (P20GM121310) from the National Institutes of Health. Quantitative densitometry analysis was conducted using the iBright analysis software. Active PAD and inactive PAD samples were normalized to the native tubulin to allow for comparison across different experiments. All values are expressed as the mean ± SEM. Means were separated using student’s *t*-test and * indicates significantly different means *p* < 0.05.

#### 4.7.2. Statistical Analysis

Graphs and statistical analysis was performed with GraphPad Prism 6.0 (GraphPad Software, San Diego, CA, USA). All experiments were independently repeated at least three times and resulting values are expressed as the mean ± SEM. Means were separated using SNK ANOVA or student’s *t*-test and * indicates significantly different means * *p* < 0.05, ** *p* < 0.01, and *** *p* < 0.001.

## Supplementary Materials

Supplementary materials can be found at https://www.mdpi.com/1422-0067/21/7/2634/s1. Table S1. Citrullinated proteins identified by LC-MS/MS. The full list of citrullinated (cit) proteins (107), proteins found in cit and control samples (35), and found only in control samples (7) using biotin-phenylglyoxal enrichment of citrullinated proteins.

## Abbreviations

JAK	Janus kinase
STAT	Signal transducer and activator of transcription
GAS	Interferon-γ-activated sequence
*Btn1a1*	Butyrophilin
*Csn2*	β-casein
CLD	Cytoplasmic lipid droplet
LC-MS/MS	Liquid chromatography with tandem mass spectrometry
Biotin-PG	Biotin-phenylglyoxal
PAD/PADI	Peptidylarginine deiminase
BB-ClA	Biphenyl-benzimidazole-Cl-amidine
PRL	Prolactin
RA	Rheumatoid arthritis
L9	Lactation day 9
PTM	Post-translational modification

## References

[1] Cherrington B.D., Morency E., Struble A.M., Coonrod S.A., Wakshlag J.J. (2010). Potential role for peptidylarginine deiminase 2 (pad2) in citrullination of canine mammary epithelial cell histones. PLoS ONE.

[2] Li G., Hayward I.N., Jenkins B.R., Rothfuss H.M., Young C.H., Nevalainen M.T., Muth A., Thompson P.R., Navratil A.M., Cherrington B.D. (2016). Peptidylarginine deiminase 3 (pad3) is upregulated by prolactin stimulation of cid-9 cells and expressed in the lactating mouse mammary gland. PLoS ONE.

[3] Cherrington B.D., Mohanan S., Diep A.N., Fleiss R., Sudilovsky D., Anguish L.J., Coonrod S.A., Wakshlag J.J. (2012). Comparative analysis of peptidylarginine deiminase-2 expression in canine, feline and human mammary tumours. J. Comp. Pathol..

[4] Darrah E., Rosen A., Giles J.T., Andrade F. (2012). Peptidylarginine deiminase 2, 3 and 4 have distinct specificities against cellular substrates: Novel insights into autoantigen selection in rheumatoid arthritis. Ann. Rheum. Dis..

[5] Witalison E.E., Thompson P.R., Hofseth L.J. (2015). Protein arginine deiminases and associated citrullination: Physiological functions and diseases associated with dysregulation. Curr. Drug Targets.

[6] Badillo-Soto M.A., Rodriguez-Rodriguez M., Perez-Perez M.E., Daza-Benitez L., Bollain Y.G.J.J., Carrillo-Jimenez M.A., Avalos-Diaz E., Herrera-Esparza R. (2016). Potential protein targets of the peptidylarginine deiminase 2 and peptidylarginine deiminase 4 enzymes in rheumatoid synovial tissue and its possible meaning. Eur. J. Rheumatol..

[7] Tilvawala R., Nguyen S.H., Maurais A.J., Nemmara V.V., Nagar M., Salinger A.J., Nagpal S., Weerapana E., Thompson P.R. (2018). The rheumatoid arthritis-associated citrullinome. Cell Chem. Biol..

[8] Khan S.A., Edwards B.S., Muth A., Thompson P.R., Cherrington B.D., Navratil A.M. (2016). Gnrh stimulates peptidylarginine deiminase catalyzed histone citrullination in gonadotrope cells. Mol. Endocrinol..

[9] Young C.H., Rothfuss H.M., Gard P.F., Muth A., Thompson P.R., Ashley R.L., Cherrington B.D. (2017). Citrullination regulates the expression of insulin-like growth factor-binding protein 1 (igfbp1) in ovine uterine luminal epithelial cells. Reproduction.

[10] Jiang Z., Cui Y., Wang L., Zhao Y., Yan S., Chang X. (2013). Investigating citrullinated proteins in tumour cell lines. World J. Surg Oncol..

[11] van Beers J.J., Schwarte C.M., Stammen-Vogelzangs J., Oosterink E., Bozic B., Pruijn G.J. (2013). The rheumatoid arthritis synovial fluid citrullinome reveals novel citrullinated epitopes in apolipoprotein e, myeloid nuclear differentiation antigen, and beta-actin. Arthritis Rheum..

[12] Senshu T., Kan S., Ogawa H., Manabe M., Asaga H. (1996). Preferential deimination of keratin k1 and filaggrin during the terminal differentiation of human epidermis. Biochem. Biophys. Res. Commun..

[13] Wang F., Chen F.F., Gao W.B., Wang H.Y., Zhao N.W., Xu M., Gao D.Y., Yu W., Yan X.L., Zhao J.N. (2016). Identification of citrullinated peptides in the synovial fluid of patients with rheumatoid arthritis using lc-maldi-tof/tof. Clin. Rheumatol..

[14] Liu X., Robinson G.W., Wagner K.U., Garrett L., Wynshaw-Boris A., Hennighausen L. (1997). Stat5a is mandatory for adult mammary gland development and lactogenesis. Genes Dev..

[15] Radhakrishnan A., Raju R., Tuladhar N., Subbannayya T., Thomas J.K., Goel R., Telikicherla D., Palapetta S.M., Rahiman B.A., Venkatesh D.D. (2012). A pathway map of prolactin signaling. J. Cell Commun. Signal..

[16] Ball R.K., Friis R.R., Schoenenberger C.A., Doppler W., Groner B. (1988). Prolactin regulation of beta-casein gene expression and of a cytosolic 120-kd protein in a cloned mouse mammary epithelial cell line. EMBO J..

[17] Anderson S.M., Rudolph M.C., McManaman J.L., Neville M.C. (2007). Key stages in mammary gland development. Secretory activation in the mammary gland: It’s not just about milk protein synthesis!. Breast Cancer Res. BCR.

[18] Nickerson S.C., Smith J.J., Keenan T.W. (1980). Role of microtubules in milk secretion--action of colchicine on microtubules and exocytosis of secretory vesicles in rat mammary epithelial cells. Cell Tissue Res..

[19] Heid H.W., Keenan T.W. (2005). Intracellular origin and secretion of milk fat globules. Eur. J. Cell Biol..

[20] McManaman J.L., Neville M.C. (2003). Mammary physiology and milk secretion. Adv. Drug Deliv. Rev..

[21] Ogg S.L., Weldon A.K., Dobbie L., Smith A.J., Mather I.H. (2004). Expression of butyrophilin (btn1a1) in lactating mammary gland is essential for the regulated secretion of milk-lipid droplets. Proc. Natl. Acad. Sci. USA.

[22] Guerin M.A., Loizzi R.F. (1980). Tubulin content and assembly states in guinea pig mammary gland during pregnancy, lactation, and weaning. Proc. Soc. Exp. Biol. Med. Soc. Exp. Biol. Med..

[23] Nickerson S.C., Keenan T.W. (1979). Distribution and orientation of microtubules in milk secreting epithelial cells of rat mammary gland. Cell Tissue Res..

[24] Rennison M.E., Handel S.E., Wilde C.J., Burgoyne R.D. (1992). Investigation of the role of microtubules in protein secretion from lactating mouse mammary epithelial cells. J. Cell Sci..

[25] Patton S., Stemberger B.H., Knudson C.M. (1977). The supression of milk fat globule secretion by clochicine: An effect coupled to inhibition of exocytosis. Biochim. Biophys. Acta.

[26] Wloga D., Joachimiak E., Fabczak H. (2017). Tubulin post-translational modifications and microtubule dynamics. Int. J. Mol. Sci..

[27] Chang X., Zhao Y., Wang Y., Chen Y., Yan X. (2013). Screening citrullinated proteins in synovial tissues of rheumatoid arthritis using 2-dimensional western blotting. J. Rheumatol..

[28] Tsai A.C., Pai H.C., Wang C.Y., Liou J.P., Teng C.M., Wang J.C., Pan S.L. (2014). In vitro and in vivo anti-tumour effects of mpt0b014, a novel derivative aroylquinoline, and in combination with erlotinib in human non-small-cell lung cancer cells. Br. J. Pharmacol..

[29] Chen C.H., Liao C.H., Chang Y.L., Guh J.H., Pan S.L., Teng C.M. (2012). Protopine, a novel microtubule-stabilizing agent, causes mitotic arrest and apoptotic cell death in human hormone-refractory prostate cancer cell lines. Cancer Lett..

[30] Bonne D., Heusele C., Simon C., Pantaloni D. (1985). 4‘,6-diamidino-2-phenylindole, a fluorescent probe for tubulin and microtubules. J. Biol. Chem..

[31] Willingham K., McNulty E., Anderson K., Hayes-Klug J., Nalls A., Mathiason C. (2014). Milk collection methods for mice and reeves‘ muntjac deer. J. Vis. Exp. JoVE.

[32] Dieterich C.M., Felice J.P., O‘Sullivan E., Rasmussen K.M. (2013). Breastfeeding and health outcomes for the mother-infant dyad. Pediatr. Clin. N. Am..

[33] Ip S., Chung M., Raman G., Chew P., Magula N., DeVine D., Trikalinos T., Lau J. (2007). Breastfeeding and maternal and infant health outcomes in developed countries. Evid Rep. Technol Assess. (Full Rep.).

[34] Lewallen D.M., Bicker K.L., Subramanian V., Clancy K.W., Slade D.J., Martell J., Dreyton C.J., Sokolove J., Weerapana E., Thompson P.R. (2015). Chemical proteomic platform to identify citrullinated proteins. ACS Chem. Biol..

[35] Horibata S., Coonrod S.A., Cherrington B.D. (2012). Role for peptidylarginine deiminase enzymes in disease and female reproduction. J. Reprod. Dev..

[36] Hagiwara T., Hidaka Y., Yamada M. (2005). Deimination of histone h2a and h4 at arginine 3 in hl-60 granulocytes. Biochemistry.

[37] Kabotyanski E.B., Huetter M., Xian W., Rijnkels M., Rosen J.M. (2006). Integration of prolactin and glucocorticoid signaling at the beta-casein promoter and enhancer by ordered recruitment of specific transcription factors and chromatin modifiers. Mol. Endocrinol..

[38] Rijnkels M., Kabotyanski E., Montazer-Torbati M.B., Hue Beauvais C., Vassetzky Y., Rosen J.M., Devinoy E. (2010). The epigenetic landscape of mammary gland development and functional differentiation. J. Mammary Gland Biol. Neoplasia.

[39] Lemay D.G., Pollard K.S., Martin W.F., Freeman Zadrowski C., Hernandez J., Korf I., German J.B., Rijnkels M. (2013). From genes to milk: Genomic organization and epigenetic regulation of the mammary transcriptome. PLoS ONE.

[40] Davey H.W., Ogg S.L., Husaini Y., Snell R.G., Korobko I.V., Mather I.H., Wilkins R.J. (1997). Structure and sequence of the bovine butyrophilin gene. Gene.

[41] Wloga D., Gaertig J. (2010). Post-translational modifications of microtubules. J. Cell Sci..

[42] Holland J.W., Thompson A. (2009). Post-translational modifications of caseins. Milk Proteins: From Expression to Food.

[43] Schmidhauser C., Bissell M.J., Myers C.A., Casperson G.F. (1990). Extracellular matrix and hormones transcriptionally regulate bovine beta-casein 5’ sequences in stably transfected mouse mammary cells. Proc. Natl. Acad. Sci. USA.

[44] Knight J.S., Subramanian V., O’Dell A.A., Yalavarthi S., Zhao W., Smith C.K., Hodgin J.B., Thompson P.R., Kaplan M.J. (2015). Peptidylarginine deiminase inhibition disrupts net formation and protects against kidney, skin and vascular disease in lupus-prone mrl/lpr mice. Ann. Rheum. Dis..

[45] Dyballa N., Metzger S. (2009). Fast and sensitive colloidal coomassie g-250 staining for proteins in polyacrylamide gels. J. Vis. Exp. JoVE.

[46] Saveliev S.V., Woodroofe C.C., Sabat G., Adams C.M., Klaubert D., Wood K., Urh M. (2013). Mass spectrometry compatible surfactant for optimized in-gel protein digestion. Anal. Chem..

[47] Scopes R.K. (1974). Measurement of protein by spectrophotometry at 205 nm. Anal. Biochem..

[48] Keller A., Nesvizhskii A.I., Kolker E., Aebersold R. (2002). Empirical statistical model to estimate the accuracy of peptide identifications made by ms/ms and database search. Anal. Chem..

[49] Searle B.C., Turner M., Nesvizhskii A.I. (2008). Improving sensitivity by probabilistically combining results from multiple ms/ms search methodologies. J. Proteome Res..

[50] Kall L., Storey J.D., MacCoss M.J., Noble W.S. (2008). Assigning significance to peptides identified by tandem mass spectrometry using decoy databases. J. Proteome Res..

[51] Nesvizhskii A.I., Keller A., Kolker E., Aebersold R. (2003). A statistical model for identifying proteins by tandem mass spectrometry. Anal. Chem..

[52] Schoenmakers T.J., Visser G.J., Flik G., Theuvenet A.P. (1992). Chelator: An improved method for computing metal ion concentrations in physiological solutions. Biotechniques.

[53] Musse A.A., Polverini E., Raijmakers R., Harauz G. (2008). Kinetics of human peptidylarginine deiminase 2 (hpad2)--reduction of ca2+ dependence by phospholipids and assessment of proposed inhibition by paclitaxel side chains. Biochem. Cell Biol..

